# Study on Effect of Surface Micro-Texture of Cemented Carbide on Tribological Properties of Bovine Cortical Bone

**DOI:** 10.3390/mi15080994

**Published:** 2024-07-31

**Authors:** Peng Shang, Bingfeng Liu, Chunhai Guo, Peijuan Cui, Zhanlin Hou, Fengbin Jin, Jianjun Zhang, Shijie Guo, Yuping Huang, Wenwu Zhang

**Affiliations:** 1 State Key Laboratory for Reliability and Intelligence of Electrical Equipment, Engineering Research Center of Ministry of Education for Intelligent Rehabilitation Device and Detection Technology, Hebei Key Laboratory of Smart Sensing and Human-Robot Interaction, School of Mechanical Engineering, Hebei University of Technology, Tianjin 300401, China; 202231205119@stu.hebut.edu.cn (B.L.); 202321202019@stu.hebut.edu.cn (F.J.); zhjjun@hebut.edu.cn (J.Z.); guoshijie@hebut.edu.cn (S.G.); 2 Ningbo Institute of Materials Technology and Engineering, Chinese Academy of Sciences, Ningbo 315201, China; zhangwenwu@nimte.ac.cn; 3 Beijing Institute of Precision Mechatronics and Controls, Beijing 100076, China; peijuan.cui@lasat.com (P.C.); zhanlin.hou@lasat.com (Z.H.); yuping.huang@lasat.com (Y.H.)

**Keywords:** micro-texture, fluid simulation, cemented carbide, bovine cortical bone, friction coefficient

## Abstract

In bone-milling surgical procedures, the intense friction between the tool and bone material often results in high cutting temperatures, leading to the thermal necrosis of bone cells. This paper aims to investigate the effect of micro-texture on the tribological properties of YG8 cemented carbide in contact with bone. The main objective is to guide the design of tool surface microstructures to reduce frictional heat generation. To minimize experimental consumables and save time, numerical simulations are first conducted to determine the optimal machining depth for the texture. Subsequently, micro-textures with different shapes and pitches are prepared on the surface of YG8 cemented carbide. These textured samples are paired with bovine cortical bone pins featuring various bone unit arrangements, and friction and wear tests are conducted under physiological saline lubrication. The experimental results indicate that the appropriate shape and pitch of the micro-texture can minimize the coefficient of friction. The parallel arrangement of bone units exhibits a lower coefficient of friction compared to the vertical arrangement. This study holds significant implications for the design and fabrication of future micro-texture milling cutters.

## 1. Introduction

Bone-milling surgery, a common procedure in orthopedic surgery, is characterized by high cutting speeds and significant heat generation due to friction between the tool and the cortical bone. The low thermal conductivity of bone materials exacerbates this issue, making it easy to cause the thermal necrosis of osteoblasts, thereby reducing the quality of the surgery [1]. Therefore, reducing the friction coefficient between the tool and the cortical bone by improving the friction state at the tool–chip interface is crucial for enhancing surgical outcomes and accelerating patient recovery [2]. Currently, most scholars have found that, in practical applications, textured friction pair surfaces with specific shapes exhibit superior tribological properties compared to smooth surfaces [3].

Hamilton [4] machined micro raised structures on a stator surface and found that these microstructures could increase the load-carrying capacity under lubricated conditions. He was the first to propose the additional fluid dynamic pressure effect. This discovery laid the theoretical foundation for subsequent research in the field of micro-textures. Etsion [5,6] developed a fluid dynamic pressure lubrication model by integrating Hamilton’s theory with Reynolds equation, thereby refining Hamilton’s original work. By optimizing the texture parameters, Etsion achieved the optimal dynamic pressure lubrication effect. Subsequently, numerous scholars, both domestic and international, have conducted extensive research on the impact of micro-textures on fluid dynamic pressure lubrication performance. Li et al. [7] designed a new kite-shaped texture and investigated the effect of the texture depth on the bearing capacity and friction coefficient. It was found that the texture exhibited the maximum bearing capacity and the minimum coefficient of friction when the dimensionless depth of the texture was 1.25. Arif et al. [8] investigated the effect of micro-groove-shaped textures on the bearing capacity of hybrid thrust bearings using numerical simulations to determine the optimum values for the groove width and depth. In a subsequent study, the effect of cavitation was considered based on the JFO boundary conditions. The results showed that the introduction of cavitation counteracted part of the negative pressure and improved the oil film bearing capacity. Zhang et al. [9] established three different shapes of groove-type textures and numerically simulated them using the finite element software FLUENT 19.2. The results showed that the sinusoidal texture provided the best hydrodynamic bearing capacity, which was consistent with subsequent friction and wear experimental results. The studies of the aforementioned scholars have demonstrated that the introduction of micro-textures enhances the fluid film bearing capacity and contributes to dynamic pressure lubrication. However, the optimal depth and area rate of these micro-textures vary under different working conditions. Therefore, further simulation studies are necessary to investigate the effects of different micro-texture sizes under water-lubricated conditions.

In addition to studying the dynamic pressure lubrication performances of textures using numerical simulations, scholars have also conducted numerous experiments to investigate the actual friction reduction effects of these textures. Zheng et al. [10] investigated the friction and wear mechanisms of three different texture shapes under dry friction conditions. They found that, in the early stage of the experiment, the textures mainly reduced the friction coefficient by collecting wear debris. In the later stage, the texture pits were filled, forming a soft–hard composite surface that exhibited a better wear resistance and lower friction coefficient. Pratap et al. [11,12] investigated the effect of micro-textures on the tribological properties of a Ti-6Al-4V hip joint implant under serum lubrication conditions. The study compared the friction coefficients between three samples with parallel aligned pit textures, staggered pit textures, and a micro grid. It was found that the grid-textured samples exhibited the lowest friction coefficients. Additionally, the parallel and staggered arrangements did not significantly influence the friction coefficients. The optimal friction reduction was achieved by all three textures under medium pitch conditions. Hou et al. [13] utilized femtosecond lasers to create micro/nano triangular textures on the rake face of a cutting tool. They conducted friction and cutting experiments to evaluate the performances of these textures. The experiments demonstrated that the triangular micro/nano textures not only reduced the cutting force by lowering the coefficient of friction between the tool and chips, but also exhibited anti-adhesion and wear reduction effects. Cheng et al. [14] machined various groove textures on the surface of YG6X cemented carbide and investigated the effects of the texture pitch and friction direction on its tribological properties. The study results showed that both the coefficient of friction and wear area of the cemented carbide surface could be reduced when the texture area rate was 15.3% or 23% and the sliding direction was perpendicular to the grooves. Chen et al. [15] investigated the effect of the pit texture on the tribological properties of the interface between stainless steel and porcine femur. Friction and wear experiments were conducted under both dry friction and water lubrication conditions. The results demonstrated that the pitted texture had a friction-reducing effect under both conditions. The larger the texture area ratio, the more pronounced the friction reduction effect, but the arrangement of the texture had little effect on the coefficient of friction. Although numerous studies on the friction-reducing properties of textures have been reported, most have focused on the metalworking field, with fewer studies conducted in the biomedical field. Additionally, the few studies in the biomedical field have not considered the effect of the anisotropic characteristics of bone material on its tribological properties. Therefore, there is still a gap in the research regarding the effect of cemented carbide surface texturization on the tribological properties of cortical bone.

In summary, this paper first investigated the influence of rhombic texture on the dynamic pressure lubrication effect of the friction pair through numerical simulations, determining the optimal texture depth. Based on this depth, micro-textures with different shapes and pitches were machined on the surface of YG8 cemented carbide. The processed texture samples were then combined with bovine cortical bone pins to form a friction pair, and reciprocating friction and wear tests were conducted. The effects of the bone unit arrangement, texture shape, and texture pitch on the friction coefficient at the contact interface were compared.

## 2. Simulation Study of Dynamic Pressure Lubrication Properties of Rhombus Texture

### 2.1. Modeling Texture Geometry

**Figure 1:**
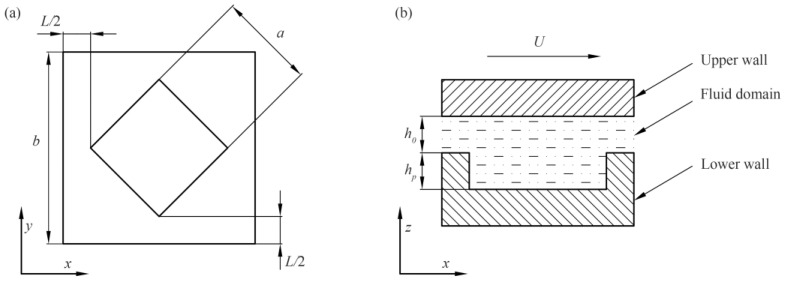
Texture geometry model. (**a**) Top view of texture cell and (**b**) sectional diagram of texture fluid domain.

Based on the group’s previous research, the rhombus texture exhibits a larger convergence wedge and divergence wedge compared to the square and circular textures. When fluid flows through the texture outlet, the pressurization effect becomes significant, clearly demonstrating the dynamic pressure lubrication mechanism of the texture. Consequently, we selected the rhombus texture for the numerical simulations. Before the fluid simulation experiments began, the texture fluid domain needed to be geometrically modeled. To save experimental time, only a single shape texture was modeled. SolidWorks was used to create rhombus texture cells with different depths and pitches to investigate the effect of these variations on the dynamic pressure lubrication performance. Based on the simulation results, the optimal texture depth for the dynamic pressure lubrication performance was determined. By neglecting the effect of curvature and considering calculation efficiency, the rhombus texture could be simplified to a single texture unit for analysis. The geometric model of the texture is shown in Figure 1.

The geometric model parameters are as follows: *L* is the pitch of the texture, with values of 30 μm, 60 μm, 90 μm, 120 μm, and 150 μm. *L*/2 represents the half pitch of the texture. The overall edge length of the cell is denoted by *b*. The texture edge length is *a*, taken as 150 μm. The moving speed of the upper wall, *U*, matches the reciprocation speed used in the subsequent friction and wear tests and was set to 0.4 m/s. The initial thickness of the liquid film, *h*_0_, was fixed at 3 μm. The depth of the texture, *h_p_*, was 5 μm, 10 μm, 15 μm, 20 μm, and 25 μm, with five values considered. For each texture pitch condition, five texture cells with different depths were constructed, resulting in a total of 25 texture fluid domain cells with different geometries. The arrangements of specific simulation experiments are shown in Table 1.

### 2.2. Mathematical Modeling of Fluid Dynamic Pressure

The Reynolds equation is one of the basic equations in the theory of dynamic pressure lubrication. It elucidates the principle of generating dynamic pressure within the liquid film situated between two friction surfaces in relative motion. The following assumptions are made:

(1) Fluid dynamic viscosity is constant. The effect of inertial forces is neglected.
(2) There is no relative sliding between the fluid and the wall. The upper wall moves at the same velocity as the fluid layer in contact with it.
(3) The fluid is a Newtonian fluid and the shear stress is proportional to the velocity.
(4) There is no fluid penetration in the film thickness direction, implying that the velocity in the z-direction is zero.

Under the fulfillment of the above assumptions, the Reynolds equation can be derived from the Navier–Stokes equation and continuity equation. The resulting form of the Reynolds equation is as shown in Equation (1).

(1) $$\frac{\partial }{\partial x}\left(\frac{h^{3}}{\mu }\frac{\partial p}{\partial x}\right)+\frac{\partial }{\partial y}\left(\frac{h^{3}}{\mu }\frac{\partial p}{\partial y}\right)=6\left[\left(U_{1}+U_{2}\right)\frac{\partial h}{\partial x}+\left(V_{1}+V_{2}\right)\frac{\partial h}{\partial y}\right]+12\frac{\partial h}{\partial t}$$

where *h* is the thickness of the liquid film; *μ* is the lubricant dynamic viscosity; *p* represents the pressure; and the velocities of the upper and lower walls of the friction pair along the *x* and *y* directions are represented by *U*_1_, *U*_2_ and *V*_1_, *V*_2_, respectively. For this study, only the motion of the wall along the *x* direction is considered, thus *V*_1_ + *V*_2_ = 0. Set *U*_1_ + *U*_2_ = *U*, where *U* is the combined velocity of the walls in the *x* direction. We specify that the velocity of the lower wall is zero, so the upper wall velocity, *U*_1_, is *U*.

Under steady-state flow conditions, the liquid film thickness does not vary with time. Consequently, Equation (1) can be simplified to obtain the pressure control equation for calculation, as shown in Equation (2).

(2) $$\frac{\partial }{\partial x}\left(\frac{h^{3}}{\mu }\frac{\partial p}{\partial x}\right)+\frac{\partial }{\partial y}\left(\frac{h^{3}}{\mu }\frac{\partial p}{\partial y}\right)=6U\frac{\partial h}{\partial x}$$

The liquid film thickness can be expressed as Equation (3).

(3) $$h=\{\begin{array}{ll} h_{0} & \left(x,y\right)\notin \Omega \\ h_{0}+h_{p} & \left(x,y\right)\in \Omega \end{array}$$

where *h*_0_ is the initial liquid film thickness; *h_p_* is the texture depth; and Ω is the texture region.

### 2.3. Boundary Condition Setting and Lubrication Performance Parameter Calculation

**Figure 2:**
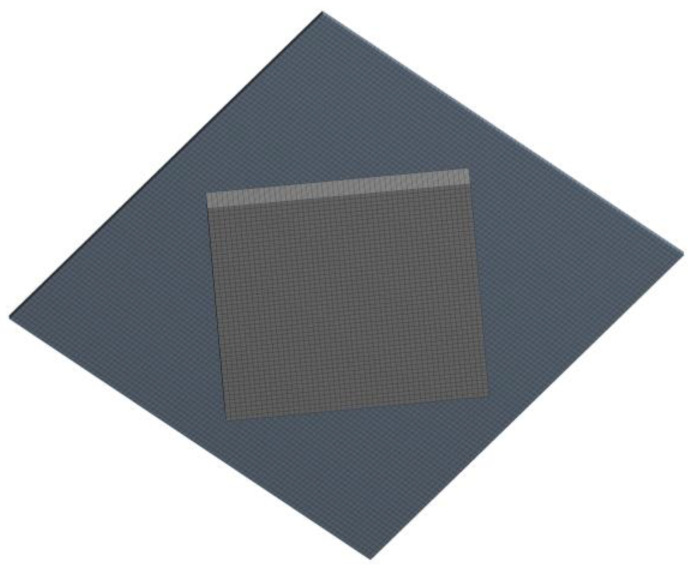
Texture fluid domain meshing image.

In this study, the FLUENT software was utilized for numerical calculations, while SolidWorks was employed to model the texture cells. The lower wall surface was fixed, and the upper wall had a velocity of *U*. The left and right boundaries were set as periodic boundary conditions along the *x*-axis direction, and the front and back boundaries were set as pressure boundary conditions along the *y*-axis direction. The density of the lubricant was 998.2 kg/m^3^ and the dynamic viscosity was 0.001 pa·s. The k-ε turbulence model was selected to describe the fluid flow state. The SIMPLEC algorithm, a pressure-coupling solver, was chosen, and the residual value was set to 10^−6^. Irrelevance validation was performed for the mesh size. The average bearing capacity increased slowly when the mesh size was less than 3 μm. To balance accuracy and computational efficiency, the mesh size was set to 3 μm. The mesh image of the fluid domain of the rhombus texture is shown in Figure 2.

The lubricating fluid flows through the texture, and due to the dynamic pressure effect, it exerts a load-bearing force perpendicular to the upper wall surface, denoted as *F_z_*. Additionally, the fluid shear motion exerts a shear force, *F_x_*, on the upper wall surface in the opposite direction of its movement. These two forces can be obtained by integrating Equation (4).

(4) $$\left\{ \begin{array}{l} F_{x}=\iint \tau dxdy\\ F_{z}=\iint pdxdy \end{array}\right.$$

The above two parameters can be obtained directly from FLUENT. To evaluate the hydrodynamic pressure lubrication performance, the average load-bearing force and friction coefficient need to be calculated through a simple conversion, as shown in Equation (5).

(5) $$\left\{ \begin{array}{l} F_{a}=\frac{F_{z}}{b^{2}}\\ f=\frac{F_{x}}{F_{z}} \end{array}\right.$$

where *F_a_* is the average bearing capacity and *f* is the coefficient of friction.

### 2.4. Effect of Texture Depth on Dynamic Pressure Lubrication Properties

**Figure 3:**
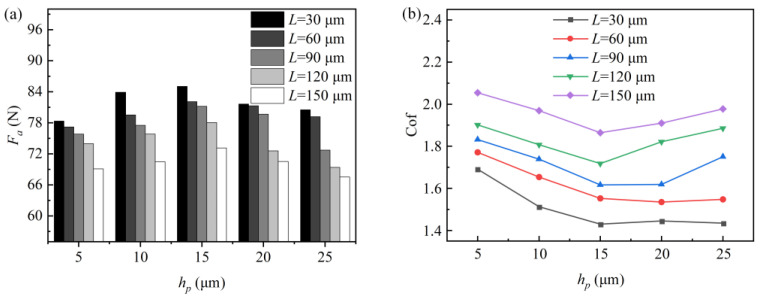
Effect of texture depth on dynamic pressure lubrication performance. (**a**) The influence of texture depth on the bearing capacity and (**b**) the effect of texture depth on friction coefficient.

The effect of the texture depth on the dynamic pressure lubrication performance of the rhombus texture is shown in Figure 3. Figure 3a,b show the plots of the rhombus texture bearing capacity *F_a_* and friction coefficient *f* with the texture depths for different pitches, respectively. It is observed that, as the texture depth increases from 5 μm to 15 μm, the bearing capacity tends to increase, while the coefficient of friction tends to decrease. When the texture depth continues to increase to 25 μm, the bearing capacity begins to decrease, and the friction coefficient for textures with a pitch of 90 μm and above starts to increase. At this stage, the trends of the bearing capacity and friction coefficient are opposite. The maximum bearing capacity and relatively low friction coefficient are achieved at a texture depth of 15 μm. However, the variation in friction coefficients for rhombus-shaped textures with pitches of 30 µm and 60 µm is smaller when the texture depth is greater than 15 µm. This is because, as the texture depth increases, the shear force exerted by the fluid on the wall decreases [16]. The friction coefficient curves exhibit a fluctuating trend when the shear force reduction and the decrease in bearing capacity are comparable.

**Figure 4:**
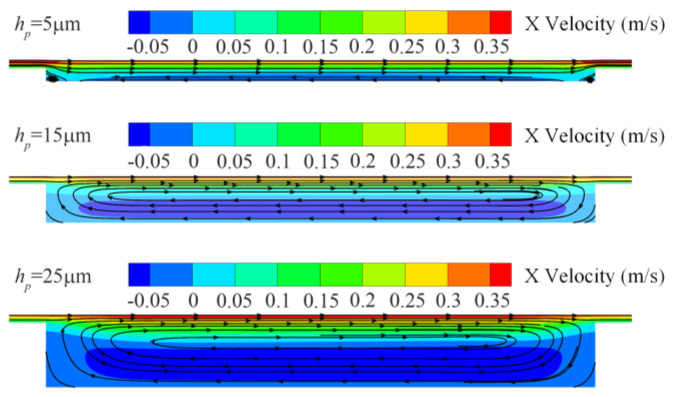
Velocity cloud map and streamline map of different depth textures in the *xz* section.

Figure 4 shows the velocity cloud and streamline diagrams of the *xz* cross-section of the texture at different texture depths. Observing the direction of the streamlines, it can be found that, at a texture depth of 5 μm, a slight countercurrent appears at the bottom of the texture. Due to the small depth, the countercurrent does not form a vortex and has little effect on the bearing capacity of the texture. At this stage, the fluid flows through the edge of the texture, and the small change in the cross-sectional area results in a less obvious wedge effect, leading to a small bearing capacity. As the depth of the texture increases to 15 μm, the backflow at the bottom of the texture becomes more pronounced and forms a large vortex. This vortex consumes part of the fluid’s kinetic energy, affecting the increase in the texture’s bearing capacity. Simultaneously, the increase in the texture depth enhances the wedge effect. Here, the positive feedback from the increased texture depth outweighs the negative feedback from the vortex formation, resulting in an increased bearing capacity [17]. However, when the texture depth increases to 25 μm, the vortex further enlarges and occupies most of the texture region. At this point, the negative effect of increased eddy currents counteracts the positive effect of the increased texture depth. Consequently, the bearing capacity of the texture decreases, and the fluid dynamic pressure lubrication performance is weakened.

In summary, the maximum load-bearing capacity and relatively low friction coefficient are achieved at a texture depth of 15 μm. The dynamic pressure lubrication of the fluid at this depth is optimal. Therefore, a processing depth of 15 μm was chosen for the subsequent preparation of the textured specimens.

### 2.5. Effect of Texture Pitch on Dynamic Pressure Lubrication Properties

**Figure 5:**
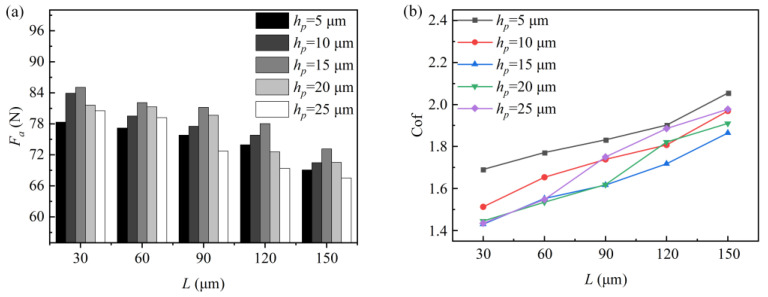
Effect of texture pitch on dynamic pressure lubrication performance. (**a**) The influence of texture pitch on the bearing capacity and (**b**) the effect of texture pitch on friction coefficient.

Figure 5 shows the graphs of the texture bearing capacity and friction coefficient with pitch. Analyzing Figure 5a,b, it can be observed that the texture bearing capacity decreases with an increasing pitch, while the friction coefficient increases. When the pitch is 30 μm, the rhombus textures with different depths exhibit the maximum bearing capacity and the minimum friction coefficient. At this pitch, the dynamic pressure lubrication performance of the texture is optimal. When the texture size is constant, increasing the pitch has a limited effect on the peak pressure of the converging and diverging wedges of the texture. However, the increase in pitch increases the overall area of the texture cell. This reduces the area share of the converging wedge relative to the overall texture cell area, gradually weakening the positive effect on the upper wall surface. Consequently, the average load-carrying capacity *F_a_* decreases and the friction coefficient increases.

## 3. Experimental System and Method

### 3.1. Experimental Equipment

Currently, common texturing techniques include EDM, micro-milling, and laser machining [18]. EDM is the use of current pulses generated by the thermal effect of melting the substrate material, forming pits on the material surface. This method is suitable for processing hard materials. However, it has the disadvantages of complex processing procedures and difficulties in controlling the shape and accuracy of the pits. Micro-milling uses a micro-milling cutter to etch micro textures onto the material surface. It has low processing costs and does not produce thermal effects on the substrate. However, the vibration generated by high-speed milling can cause deviations in texture size. Laser processing is a non-contact method that uses laser focusing to irradiate the surface of the material to melt and gasify the material. This technique offers a simple processing procedure, high efficiency, and precise control of the texture size. Its disadvantage is that it will produce a heat-affected zone, thereby forming defects such as burrs and recast layers, which will reduce the surface quality.

**Figure 6:**
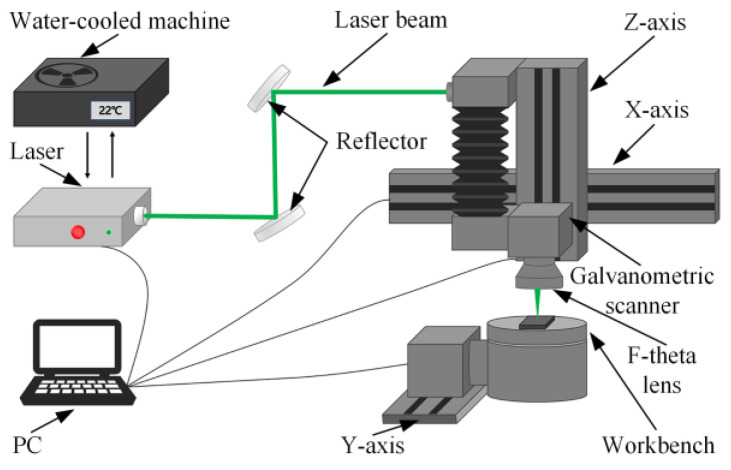
Schematic diagram of laser processing principle.

Laser processing mainly includes nanosecond, picosecond, and femtosecond lasers. Among these, femtosecond laser processing has the shortest pulse width, resulting in the smallest heat-affected zone and the highest precision in texture dimension control. Therefore, in this study, a five-axis femtosecond laser processing platform was used to create the micro-textures on the surface of the YG8 cemented carbide. The laser processing principle is illustrated in Figure 6. The laser emits infrared light, which is transmitted to the galvanometric scanner and f-theta lens via a fixed optical path. The scanning path and speed are configured in the EzCad 2.0 software, which then controls the galvanometer’s rotation, allowing for precise control over the texture’s shape and position. The main parameters of the femtosecond laser are detailed in Table 2.

**Figure 7:**
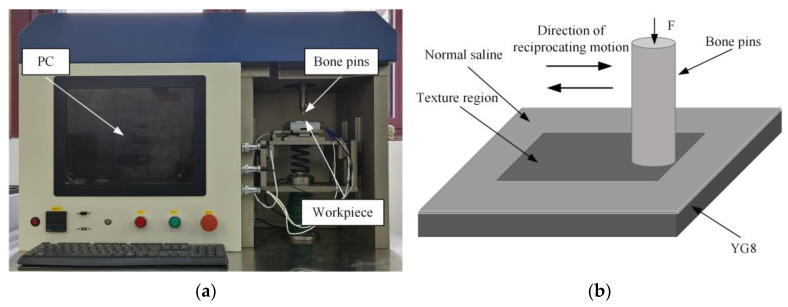
MZF-1 reciprocating friction tester and principle schematic diagram. (**a**) MZF-1 reciprocating friction and wear tester and (**b**) schematic diagram of reciprocating friction principle.

Friction and wear tests were conducted using an MZF-1 reciprocating friction and wear tester, as shown in Figure 7. Cylindrical bone pins were bolted to the upper part of the sample. The textured sample was secured to the working table with a special fixture using bolt fastening. The motor spindle rotated to drive the slider linkage mechanism, enabling reciprocating motion. The speed of the pin movement could be adjusted by changing the reciprocating stroke and frequency. The table was equipped with force sensors in the vertical and horizontal directions to control the applied load and to collect friction force data.

### 3.2. Sample Preparation

**Figure 8:**
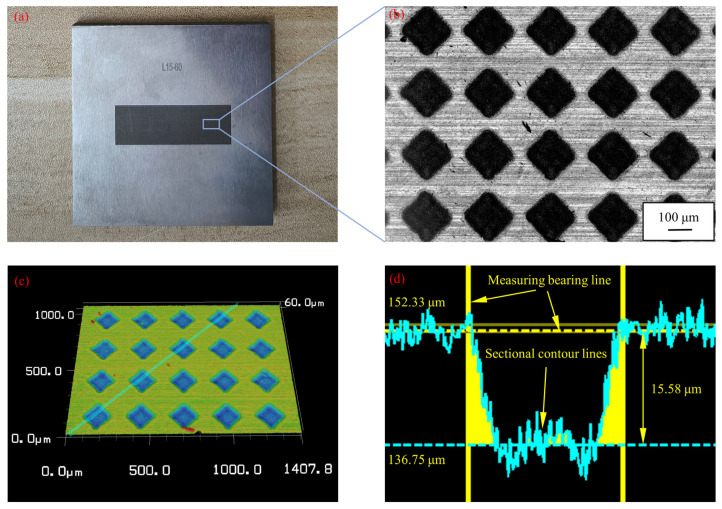
Rhombus textured sample diagram. (**a**) Textured sample; (**b**) the surface morphology of rhombus textured sample; (**c**) three-dimensional morphology of rhombus texture samples; and (**d**) measurement of rhombus texture size.

The experimental sample material was YG8 cemented carbide. Cemented carbide milling cutters are frequently utilized in bone-cutting surgery and bone-milling performance research due to their high hardness and wear resistance [19,20]. Consequently, cemented carbide was selected as the material for processing micro-textures, forming a friction pair with cortical bone. The cemented carbide was machined into square pieces measuring 50 mm × 50 mm × 5 mm using wire cutting. The upper and lower surfaces of the sample were flat ground to meet the surface roughness requirements for laser processing. Before laser machining, the samples were ultrasonically cleaned with anhydrous ethanol for 15 min. Considering the processing efficiency and the reciprocating stroke of the friction and wear tester, the texture processing location was set to a rectangular area of 30 mm × 10 mm in the center of the sample. Based on the simulation results from the previous chapter, the micro-texture machining depth was determined to be 15 μm. The laser processing parameters required for texture machining on cemented carbide surfaces to a depth of 15 μm were determined by laser processing tests, as shown in Table 3. Currently, there are few reports on the effect of micro-texture on the tribological properties of the interface between cemented carbide and bio-bone materials. To fill this gap, the present study focused on the micro-texture of basic symmetric geometries. The final texture processing shapes were determined to be square, circular, and rhombus. These are the three most representative shapes. Many bionic shape designs are derived from these shapes. The texture pitch was set at 30 μm, 60 μm, 90 μm, 120 μm, and 150 μm. The surface morphology and texture depth of the samples were measured using a confocal microscope, as shown in Figure 8. The measured depth of the rhombus texture was 15.58 μm, and the length of the texture edge was 148.83 μm, which met the processing requirements.

**Figure 9:**
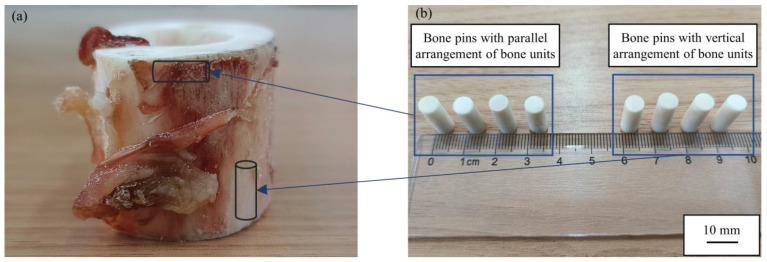
Bone pins preparation. (**a**) Fresh bovine rod bone and (**b**) different arrangements of bone unit bone pins.

The pin material used was bovine cortical bone, which closely resembles the mechanical properties of human bone. Given that bone is a fiber matrix composite, its mechanical properties exhibit anisotropy, leading to some differences in its tribological properties in different directions. To explore the tribological properties of bone materials in different orientations and simulate the friction state of the tool–bone interface in various cutting directions, two groups of bone pins with different bone unit arrangements were prepared. One group of bone pins was prepared using fresh bovine rod bones along the direction of parallel bone unit fibers, ensuring that the bone units were perpendicular to the contact surface between the bone pin and the cemented carbide. Another set of bone pins was prepared along the direction perpendicular to the bone unit fibers to ensure that the bone units on the upper and lower surfaces of the bone pins were arranged in parallel. As shown in Figure 9, the diameter of each bone pin was approximately 5.8 mm, and the length was about 15 mm.

### 3.3. Experimental Methods

The aim of this experiment was to investigate the effects of the texture shape, texture pitch, and bone unit arrangement on the tribological properties of the contact interface between cemented carbide and bovine cortical bone. To control the experimental costs and reduce the number of experiments, we determined the texture depth value with the best hydrodynamic lubrication performance through simulation. In the simulation process, the moving velocity of the upper wall was constant. The speed of the pin changed constantly during the reciprocating friction test. There were some differences between the two. However, when the reciprocating friction and wear tester had a large enough stroke and a low enough reciprocating frequency, we could approximately assume that the pin was moving at a constant speed. Additionally, we chose symmetric texture shapes to accommodate the constant changes in the pin’s moving direction during the test. Finally in our previous study, the optimal texture depth value had little correlation with the wall movement speed. In summary, we could approximately assume that the best depth results obtained by the simulations were suitable for the friction and wear tests. To further verify the reliability of the simulation results, we compared the friction coefficients obtained by the simulations and experiments under the same conditions and comparatively analyzed the trends of the two friction coefficients with the variation in the rhombus texture pitch.

The specific experimental arrangements were as follows. Saline was used as a lubricant. The lubricant was supplied non-continuously during the test. A 3 mL drop of saline was pipetted onto the upper surface of the sample before the start of each test to provide lubrication to the friction interface. According to the speed of the tip of the tool and the milling force during the bone milling, the load was set to 30 N and the reciprocating speed was set to 0.4 m/s. The reciprocating speed was controlled by the reciprocating stroke and the reciprocating frequency, which were set to 40 mm and 10 Hz. Due to the significant hardness difference between the bone material and cemented carbide, the bone pins wore out faster during the test. If the test time was too long, dislodged bone chips and saline could have formed a suspension, affecting the lubrication state between the two friction parts. Conversely, if the test time was too short, the friction coefficient may not have stabilized, failing to accurately reflect the tribological performance between the two surfaces. Therefore, the test duration was set to 10 min. The specific experimental program is shown in Table 4. Experiments 1 and 17 served as no-texture control groups, denoted by NT.

## 4. Experimental Results and Discussion

### 4.1. Effect of Texture Pitch on the Coefficient of Friction

**Figure 10:**
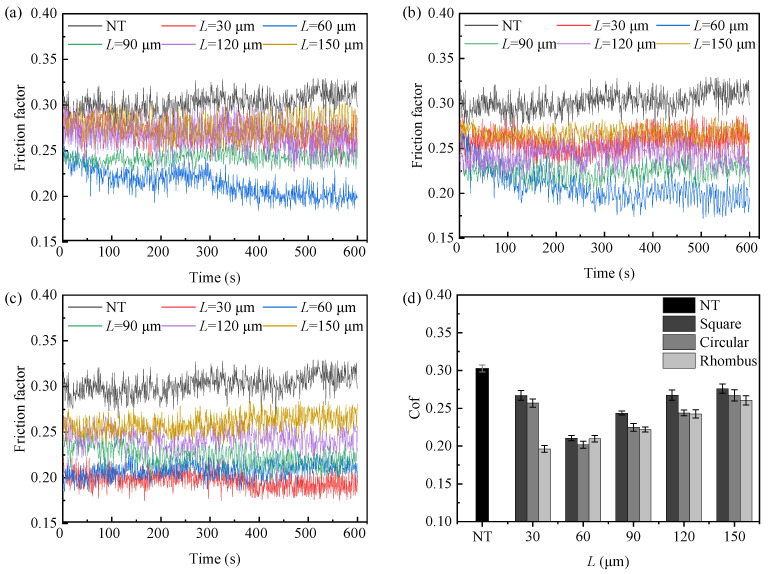
The effect of texture pitch on friction coefficient. (**a**) Friction coefficient curve of square texture; (**b**) friction coefficient curve of circular texture; (**c**) friction coefficient curve of rhombus texture; and (**d**) average friction coefficient.

The friction and wear tests were conducted on cemented carbide samples numbered 1–16. A total of three tests were repeated for each sample under the same conditions. The average friction coefficient was obtained by averaging the results of the three tests. The single dynamic friction coefficient curves and the average friction coefficients of the corresponding samples are shown in Figure 10. From the figure, it can be observed that, when the friction time was less than 100 s, the contact surface between the bone pin and cemented carbide was in the running-in stage, resulting in significant fluctuations in the friction curve. After 100 s, the test entered the stable wear stage, where the friction coefficient became relatively stable. The average friction coefficients for different textured samples were calculated from 100 to 600 s, based on the above analysis. The results are shown in Figure 10d. The average friction coefficient of the non-textured sample was 0.302. Compared to the non-textured sample, all textured samples exhibited reduced friction. This friction coefficient reduction can be attributed to the texture’s ability to store lubricant and provide secondary lubrication. Additionally, the presence of texture pits generated fluid dynamic pressure, contributing to a dynamic pressure lubrication effect [21]. The experimental results indicated that introducing texture could effectively improve the friction state of the tool–bone contact interface, thereby reducing frictional heat generation and the probability of the thermal necrosis of bone cells. Among the textured samples, the rhombus texture with a 30 μm pitch demonstrated the lowest friction coefficient of 0.197, achieving a friction reduction of 34.77%.

Analyzing Figure 10, the friction coefficient of the rhombus-textured sample showed an increasing trend with an increasing pitch, with the lowest friction coefficient observed at a texture pitch of 30 μm. As pitch increased, the texture area rate decreased, reducing the texture’s ability to store wear debris. The excess wear debris entered the friction interface, which adversely affected the friction state and increased the friction coefficient. From a fluid dynamic pressure lubrication performance perspective, the bearing capacity decreased as the pitch increased. A larger pitch also destabilized the lubricant film, resulting in a higher friction coefficient. Additionally, an increased pitch enlarged the contact area between the friction surfaces, further increasing the friction.

**Figure 11:**
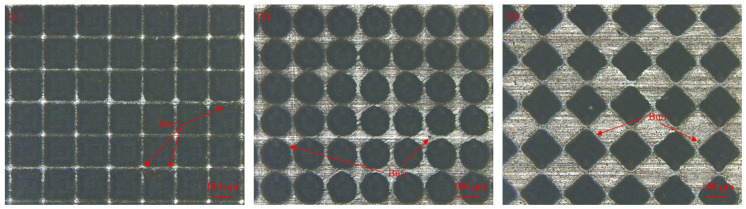
Surface morphology of textured samples with a pitch of 30 μm. (**a**) Surface morphology of square-textured samples; (**b**) surface morphology of circular-textured samples; and (**c**) surface morphology of rhombus-textured samples.

For square- and circular-textured samples, the friction coefficient initially decreased and then increased with an increasing pitch, with the lowest coefficient at a 60 μm texture pitch. This behavior was due to defects like burrs and a recast layer formed around the texture contour during the femtosecond laser ablation process [22], as shown in Figure 11. These defects increased the surface roughness of the textured sample. In the case of a pitch of 30 microns, the texture area rates of the square and circular textures were higher, which were calculated to be 69.4% and 54.5%, respectively, resulting in a smaller contact area of the friction pair, and the textured sample was greatly affected by laser processing defects. The increase in roughness accelerated the wear rate of the bone pins. The texture area filled quickly with bone chips, reducing the texture’s ability to capture debris and increasing the friction coefficient. In actual cutting processes, an increased roughness raises cutting forces, heightening the risk of bone cracks and impacting on postoperative recovery. When the texture pitch was greater than or equal to 60 μm, the texture area rate decreased, diminishing the impact of machining defects. Similar to the rhombus texture, the friction coefficient rose with an increasing texture pitch.

**Figure 12:**
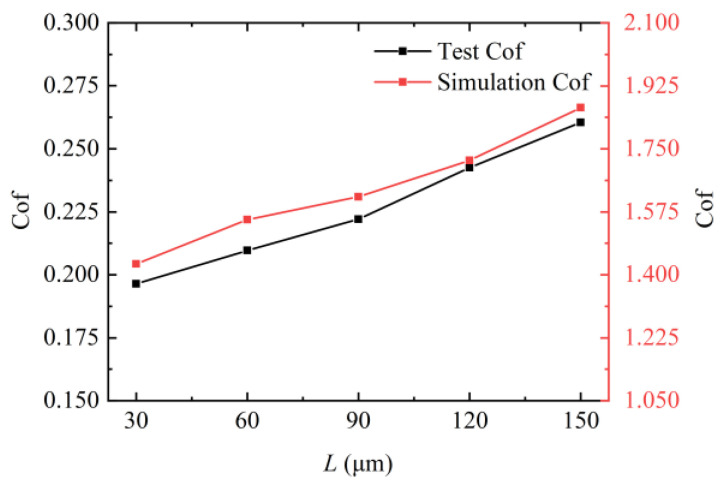
Comparison of simulation Cof and test Cof.

Figure 12 compares the simulation Cof with the tested Cof for different pitch rhombus textures. The simulated friction coefficient was found to be larger than the tested friction coefficient. This discrepancy arose because the fluid simulation analyzed only a single texture unit and was conducted under ideal conditions, considering only the effects of the fluid bearing force and shear force on the friction coefficient. This left a certain gap between the simulation conditions and the actual working conditions. However, the trend in the friction coefficient was consistent between the two. It was shown that the simulation results can partially verify the friction test results.

### 4.2. Effect of Texture Shape on the Coefficient of Friction

**Figure 13:**
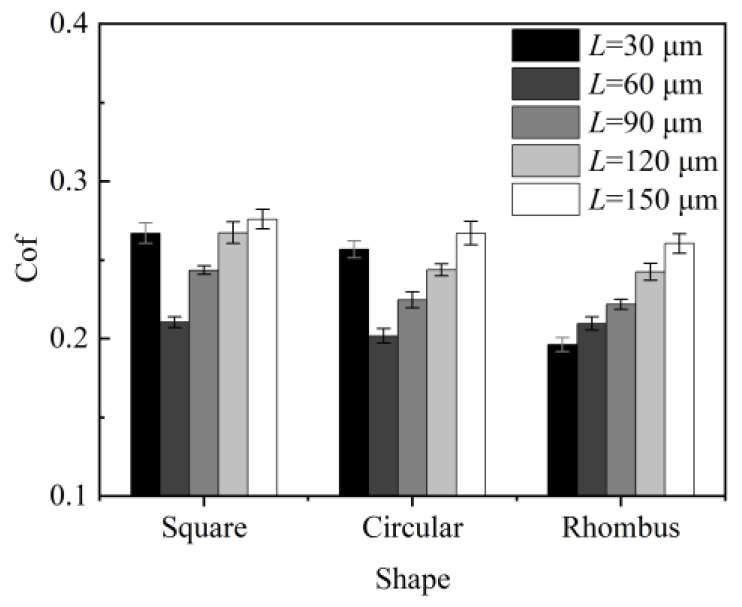
The effect of texture shape on friction coefficient.

Figure 13 illustrates the friction coefficient comparison of three samples with different texture shapes. Combined with Figure 10, it is evident that the friction coefficients of the rhombus-textured samples were lower than those of the square- and circular-textured samples at a pitch of 30 μm. Figure 11 illustrates the surface morphology of the three textured sample shapes. Spillover edges formed at the edges of the texture due to the laser ablation of the material. Additionally, some of the molten slag spilled onto the surface, resulting in the formation of a small amount of burrs. These issues contributed to an increase in the surface roughness of the sample. Observing Figure 11, the area rate of the rhombus texture was smaller than that of the square and circular textures in the 30 μm pitch condition. Therefore, it was less affected by femtosecond laser defects and the friction coefficient was the lowest. At a 60 μm pitch, the friction coefficients of the three textured samples were similar, with the circular texture having a slightly smaller coefficient. This was due to the smooth transition of the edges of the circular texture, which was less prone to stress concentrations, leading to a lower friction coefficient. When the texture pitch was 90 μm and above, the square-textured samples exhibited the highest friction coefficient, the rhombus-textured samples the lowest, and the circular-textured samples had a coefficient close to that of the rhombus. Observing the wear rate of the bone pins, the fastest wear occurred when they were ground against the square-textured samples. This was because the sharp edges of the square texture exerted a plowing effect on the bone pins, increasing the friction force.

Analyzing the hydrodynamic pressure lubrication performance, the square texture lacked convergence, resulting in its inability to develop a high fluid dynamic pressure. In contrast, the rhombus texture, with its largest converging wedge, allowed the lubricant flowing through the texture outlet to develop a higher peak pressure, enhancing the dynamic pressure lubrication performance [23]. Consequently, the rhombus texture provided the best friction reduction effect. The circular texture, with its smooth edges, caused less wear on the bone pins and featured a convergent shape. These factors made the anti-friction effect of the circular texture second only to the rhombus texture and significantly better than the square texture. However, in actual cutting processes, the sharp edges of the square texture may accelerate chip breakage. The broken chips can carry away part of the heat, thus improving the heat dissipation effect.

### 4.3. Effect of Bone Unit Arrangement on the Coefficient of Friction

**Figure 14:**
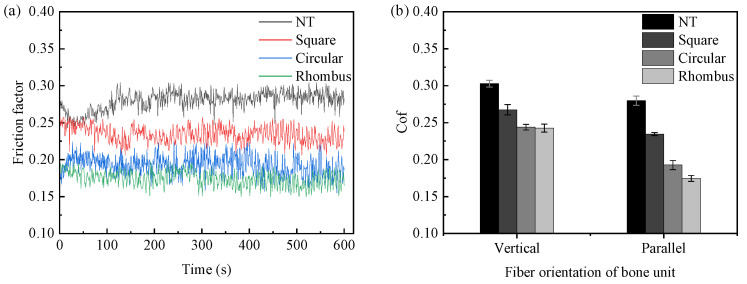
The influence of bone unit arrangement direction on friction coefficient. (**a**) Friction coefficient curve of bone pin with parallel arrangement of bone units and (**b**) average friction coefficient.

The bone pins with a parallel arrangement of bone units were replaced by bone pins with a vertical arrangement of bone units, and friction and wear experiments were conducted. The dynamic friction coefficient curves obtained are shown in Figure 14a. Figure 14b illustrates the comparison of the average friction coefficients between the two types of bone pins. From the diagrams, it is evident that the fluctuation of the dynamic friction coefficient curve is similar to that before the replacement of the bone pin. After the friction time exceeds 100 s, the curves basically enter the stable friction state. When the bone units are arranged in parallel, the friction coefficients of both the non-textured and textured samples were reduced compared to the vertical arrangement of bone units. Comparing the friction coefficients of different textured samples, it is observed that the rhombus texture consistently outperformed the circular texture, and the circular texture was better than the square texture. This demonstrates that the anisotropy of the bone material did not affect the friction reduction effect of the texture.

**Figure 15:**
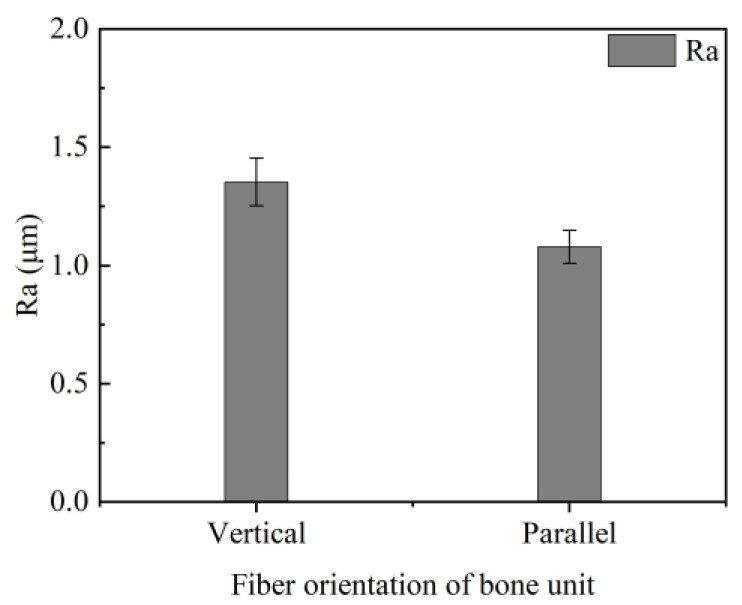
Roughness of bone pins for different bone unit arrangements.

Bone material, unlike metallic materials, is a non-homogeneous anisotropic fiber matrix composite consisting of bone units and a bone matrix [24]. The bone units are arranged along the long axis of the bone. This arrangement causes the friction and wear properties of cortical bone to vary in different directions. When the bone units are arranged vertically, the surface of the bone pin in contact with the textured sample becomes rougher compared to when the bone units are arranged in parallel. We measured the roughness of two bone pins surfaces using a non-contact profilometer, as shown in Figure 15. It can be seen that the roughness of bone pins with a vertical arrangement of bone units was higher than that of bone pins with a parallel arrangement of bone units. This was because the fibers of vertically aligned bone units exhibited fiber curl on the surface, increasing the roughness. During testing, these fibers tended to embed in the texture. The edges of the texture cut into the bone unit fibers, increasing the friction and, consequently, the coefficient of friction [25]. Additionally, bone chips formed by the wear of bone pins during the test were observed. It was found that the number of bone chips from bone pins with a parallel arrangement of bone units in the lubricant was significantly less than that from bone pins with a vertical arrangement of bone units with the same test duration. This indicates that bone pins with a parallel bone unit arrangement are less prone to wear, allowing the texture to capture abrasive debris for a longer period and reducing the effect of bone chips on friction. Moreover, bone pins with vertically arranged bone units wear faster and develop microcracks on the surface. These microcracks further increase the roughness of the pin surface.

**Figure 16:**
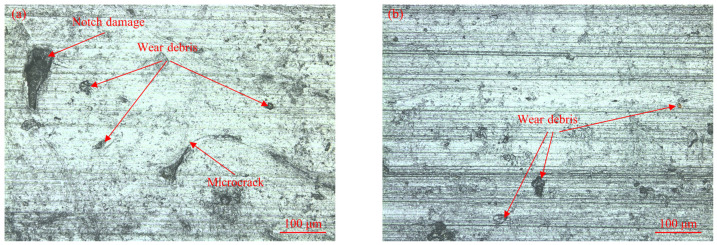
Surface morphology of different bone pins. (**a**) Surface morphology of bone pins with vertical arrangement of bone units and (**b**) surface morphology of bone pins with parallel arrangement of bone units.

As shown in Figure 16, we analyzed the surface morphology of the two bone samples after the friction test. Both bone samples exhibited relatively severe abrasive wear, with some abrasive debris remaining on the surface. This was because the hardness of cemented carbide is much higher than that of the bone material. Compared to the bone pin with a parallel arrangement of bone units, the bone pin with a vertical arrangement of bone units showed some crater damage. This damage may have resulted from the spalling of the bone units due to adhesive wear. Additionally, rapid wear under load led to the formation of microcracks on the surface of the bone pins.

## 5. Conclusions

To improve the friction state of the tool–chip interface during bone cutting surgery, thereby reducing the tool–bone contact friction coefficient and cutting heat generation, this study investigated the effects of different micro-textures on the tribological properties of the contact interface between cemented carbide and bovine cortical bone. Firstly, a numerical simulation of the rhombus-shaped texture was conducted to determine the texture depth with the optimal dynamic pressure lubrication performance. Subsequently, textured samples with various shapes and pitches were prepared based on this depth. Bone pins with different fiber arrangements of bone units were prepared along different directions of the bovine bone. Finally, friction and wear tests were conducted. The experimental results were analyzed, leading to the following conclusions:

(1) The bearing capacity showed a tendency of increasing and then decreasing with an increase in the texture depth. The maximum bearing capacity and a relatively low friction coefficient were observed at a texture depth of 15 μm. In this case, the wedge effect caused by the increased texture depth counteracted the negative effect of vortex increase. Increasing the texture pitch weakened the pressurization effect of the converging wedge on the entire upper wall, resulting in a lower bearing capacity and higher friction coefficient. Consequently, a texture depth of 15 μm was identified as optimal.
(2) With an increase in the pitch, the contact area of the friction pair increased. This weakened the dynamic pressure lubrication performance and reduced the ability to capture abrasive debris, leading to an increase in the friction coefficient of the diamond texture. The square- and circular-textured samples were affected by laser processing defects, which increased the contact surface roughness at a pitch of 30 μm. A recasting layer produced a plowing effect on bone needles, resulting in increased friction and higher friction coefficients. The smallest friction coefficient was observed at a pitch of 60 μm. Compared to the other textures, the rhombus texture exhibited the largest convergence wedge and the best dynamic pressure lubrication. The rhombus-shaped textured sample with a pitch of 30 μm reduced the coefficient of friction by 34.77% compared to the non-textured sample, demonstrating the best friction reduction effect.
(3) Regardless of the bone unit arrangement, micro-textures reduced friction. However, the tribological properties of the bone pins differed between the two bone cell arrangements. The vertical arrangement had a greater roughness on the contact surface of the bone pins with cemented carbide compared to the parallel arrangement. This increased roughness accelerated the wear of the bone pins and made the surface of the bone pins more susceptible to microcracks under load. Consequently, bone pins with vertically aligned bone units exhibited a higher coefficient of friction.
(4) Appropriate texture parameters can significantly reduce the friction coefficient between cemented carbide and bone contact surfaces. Reducing the contact friction coefficient can inhibit the frictional heat generation from chips and reduce the cutting force. In turn, the purpose of reducing thermal damage to bone cells and microcracks on the bone surface can be achieved. The results of this experiment provide valuable guidance for designing the surface microstructures of surgical instruments.

## Figures and Tables

**Table 1:** Arrangement sof simulation experiments.

Experiment Number	Texture Pitch (μm)	Texture Depth (μm)
1	30	5
2	30	10
3	30	15
4	30	20
5	30	25
6	60	5
7	60	10
8	60	15
9	60	20
10	60	25
11	90	5
12	90	10
13	90	15
14	90	20
15	90	25
16	120	5
17	120	10
18	120	15
19	120	20
20	120	25
21	150	5
22	150	10
23	150	15
24	150	20
25	150	25

**Table 2:** Laser main parameters.

Parameter	Numerical Value
Laser type	femtosecond pulsed fiber laser
Laser model	HR-Femto-IR-200-35, Huaray, Wuhan, China
Laser spot size (μm)	35
Pulse width (fs)	317
Repeat frequency rate (khz)	175
Wavelength (nm)	1030

**Table 3:** Laser processing parameters.

Parameter	Numerical Value
Single pulse energy (μJ)	15.43
Scanning speed (mm/s)	400
Scan times	3

**Table 4:** Experiment scheme.

Experiment Number	Texture Shape	Texture Pitch (μm)	Arrangement of Bone Units
1	NT	0	Vertical-arranged
2	Square	30	Vertical-arranged
3	Square	60	Vertical-arranged
4	Square	90	Vertical-arranged
5	Square	120	Vertical-arranged
6	Square	150	Vertical-arranged
7	Circular	30	Vertical-arranged
8	Circular	60	Vertical-arranged
9	Circular	90	Vertical-arranged
10	Circular	120	Vertical-arranged
11	Circular	150	Vertical-arranged
12	Rhombus	30	Vertical-arranged
13	Rhombus	60	Vertical-arranged
14	Rhombus	90	Vertical-arranged
15	Rhombus	120	Vertical-arranged
16	Rhombus	150	Vertical-arranged
17	NT	0	Parallel-arranged
18	Square	120	Parallel-arranged
19	Circular	120	Parallel-arranged
20	Rhombus	120	Parallel-arranged

## Data Availability

The original contributions presented in the study are included in the article, further inquiries can be directed to the corresponding author.

## References

[1] Tian H., Dang X., Meng D., Tian B., Li J. (2023). Influence of drilling parameters on bone drilling force and temperature by FE simulation and parameters optimization based Taguchi method. Alex. Eng. J..

[2] Lian Y., Chen X., Xie C., Long Y., Lin F., Zhou W., Chu X. (2023). Cooling and Crack Suppression of Bone Material Drilling Based on Microtextured Bit Modeled on Dung Beetle. Chin. J. Mech. Eng..

[3] Huang Y., Zhong L., Wang G., Wei G., Peng S. (2021). Research status and progress of surface texture lubrication and friction reduction. Surf. Technol..

[4] Hamilton D.B., Walowit J.A., Allen C.M. (1966). A Theory of Lubrication by Microirregularities. J. Basic Eng..

[5] Etsion I., Burstein L. (1996). A Model for Mechanical Seals with Regular Microsurface Structure. Tribol. Trans..

[6] Etsion I., Halperin G. (2002). A Laser Surface Textured Hydrostatic Mechanical Seal. Tribol. Trans..

[7] Li P., Zhang F., Zhang H., Wang T., Wang Q., Qiao W. (2023). Lubrication performance of kite-shaped microtexture under hydrodynamic lubrication. Tribol. Int..

[8] Arif M., Kango S., Shukla D.K. (2021). Investigating the effect of different slip zone locations on the lubrication performance of textured journal bearings. Ind. Lubr. Tribol..

[9] Zhang N., Yang F., Jiang F., Zhang Y., Liu G. (2020). Investigation of tribological performance of micro-groove textured cemented carbide surfaces. Surf. Eng..

[10] Zheng G., Lai F., Lin Y., Yu J., Ren Z. (2023). Study on the Friction-Reducing Mechanisms of Surface Texture Cemented Carbide under Dry Sliding. J. Mater. Eng. Perform..

[11] Pratap T., Patra K. (2020). Tribological performances of symmetrically micro-textured Ti-6Al-4V alloy for hip joint. Int. J. Mech. Sci..

[12] Pratap T., Patra K. (2018). Mechanical micro-texturing of Ti-6Al-4V surfaces for improved wettability and bio-tribological performances. Surf. Coat. Technol..

[13] Hou Q., Yang X., Li D., Cheng J., Wang S., Xiao J., Li W. (2022). Tribological performance of hydrophobic and micro/nano triangle textured rake face of cutting tools. Appl. Surf. Sci..

[14] Cheng H., Zhou F., Fei Z. (2023). Dry Friction Properties of Friction Subsets and Angle Related to Surface Texture of Cemented Carbide by Femtosecond Laser Surface Texturing. Coatings.

[15] Chen J., Zheng Q., Hu Y. (2019). Effect of dimple micro-texture on friction properties of 4Cr13 stainless steel/cortical friction pair. Tool Eng..

[16] Yin H., Yang J., Gu Q. (2024). Numerical study on the hydrodynamic lubrication performance improvement of bio-inspired peregrine falcon wing-shaped microtexture. Tribol. Int..

[17] Liu W., Ni H., Chen H., Wang P. (2019). Numerical simulation and experimental investigation on tribological performance of micro-dimples textured surface under hydrodynamic lubrication. Int. J. Mech. Sci..

[18] Chen K., Yang X., Zhang Y., Yang H., Lv G., Gao Y. (2021). Research progress of improving surface friction properties by surface texture technology. Int. J. Adv. Manuf. Technol..

[19] Liao Z., Axinte D., Gao D. (2019). On modelling of cutting force and temperature in bone milling. J. Mater. Process. Technol..

[20] Chen Q.-S., Dai L., Liu Y., Shi Q.-X. (2020). A cortical bone milling force model based on orthogonal cutting distribution method. Adv. Manuf..

[21] Jing X., Zhai Q., Zhang D., Zheng S., Jaffery S.H.I., Wang F. (2022). Wettability and frictional properties on zirconia surfaces irradiated by femtosecond laser. Colloids Surfaces A Physicochem. Eng. Asp..

[22] Yang Q., Zhang H., Zhou W. (2019). Surface incubation effect of carbide yg6 induced by femtosecond laser. Acta Photonica Sin..

[23] Cui Z., Guo Z., Yuan C. (2020). Influence of different rhombic surface textures on the tribological performance of water-lubricated bearings. Mater. Express.

[24] Shang P., Zhang H., Liu X., Yang Z., Liu B., Liu T. (2023). Cutting-Force Modeling Study on Vibration-Assisted Micro-Milling of Bone Materials. Micromachines.

[25] Li W., Zhang Z. (2019). The effect of micro-pits texture on the coefficient of friction between wood and cemented carbide. Sci. Silvae Sin..

